# The Relationship between Anxiety Symptoms and Perceived Quality of Life among Caregivers of Children with Autism Spectrum Disorder in the Amazon

**DOI:** 10.3390/ijerph21050545

**Published:** 2024-04-26

**Authors:** Rayanne Vieira da Silva, Manuela Maria de Lima Carvalhal, Daniela Lopes Gomes

**Affiliations:** 1 Nucleus of Behavior Theory Research, Federal University of Pará, Belém 66087-110, Brazil; rayanne.vs@hotmail.com; 2 Institute of Health Sciences, Federal University of Pará, Belém 66075-110, Brazil; manuela.carvalhall@gmail.com

**Keywords:** quality of life, anxiety, autism, caregivers

## Abstract

The relationship between anxiety symptoms and perceived quality of life among caregivers of children with autism was verified. To assess perceived quality of life, the Short Form Healthy Survey Item was used; perception of anxiety symptoms was assessed using the Beck Anxiety Inventory. Eighty caregivers of children with autism participated, with 68.7% of caregivers being mothers. Of the total number of caregivers, 68.8% had a severe level of anxiety. Regarding perceived quality of life, they demonstrated greater impairment in limitation due to physical aspects, limitation due to emotional aspects, vitality, and pain. Caregivers with severe anxiety levels had a worse quality of life in the domains of pain (*p* = 0.012), social aspects (*p* < 0.001), limitation due to emotional aspects (*p* = 0.001), and mental health (*p* < 0.001). However, in the functional capacity domain, caregivers with a moderate level of anxiety had a better physical capacity score (*p* = 0.001). There was a negative correlation between the general anxiety score and the general physical (*p* = 0.029) and general emotional components of perceived quality of life (*p* < 0.001). It was found that caregivers of children with ASD have a high level of anxiety, which is a predictor of the perception of a worse quality of life.

## 1. Introduction

Autism spectrum disorder (ASD) is a pervasive neurodevelopmental disorder characterized by atypical development in social communication and social interactions across different domains, along with restricted, repetitive, and stereotyped patterns of behavior, interests, or activities [1]. In Brazil, there are no official prevalence figures for ASD; however, according to the Centers for Disease Control and Prevention [2], in 2020, the general prevalence in the United States is 1 in every 36 8-year-old children.

Children diagnosed with ASD have an increased need for attention from caregivers compared to neurotypical children. The amount of attention given to the child depends on the child’s level of independence. Furthermore, it is recommended that the child and caregiver undergo early monitoring to help manage and minimize the symptoms of the disorder, through individual and collective interventions [3].

The role of caring for a person with special needs can generally be performed by a family member or another person, whether paid or unpaid. Some studies have already described the consequences faced by caregivers, such as responsibility for care, social isolation, anxiety, and emotional burden, among others, and these lead to a decrease in quality of life. The literature shows that, generally, a family member of the child is dedicated to the care provided, with the mother being primarily responsible for this task. Depending on the child’s level of independence, the caregiver must give up their occupations such as work, study, and even social relationships to dedicate themselves to the demands and responsibilities of the child [4].

According to existing research, being the main caregiver of a child with ASD, especially when there is low family and social support, can contribute a significant emotional impact when compared to caregivers of children with typical development [5,6]. Additionally, studies show that parents of children with ASD experience higher levels of depression, stress, and anxiety [7,8] and a greater impact on their quality of life [9,10]. Many theories address the family structure and its impact on the care of neurodivergent children, such as the ecological model, in which children with different types of disabilities can develop through the interaction of their skills with a responsive context [11]. But the burden that caring for children with ASD can cause still needs more studies.

In the study of Vernhet et al. [9], which aimed to compare the perceptions of mothers and fathers on the impact of autism spectrum disorder on their quality of life, mothers perceived a greater negative impact than fathers on their global quality of life, being an alert to parents’ needs in terms of social and educational support.

Musseti et al. [12] mention that several variables are associated with lower quality of life among parents of children and adolescents with ASD, including parental characteristics (being a mother, parental mental health problems, maladaptive parental coping strategies, and low parental self-efficacy), child characteristics (behavioral problems, emotional problems, level of ASD support, and age of the child), and contextual factors (low employment status, low family income, low availability of social and professional support, and lack of participation in health promotion activities).

According to Hayes and Watson [13], the main factor that will contribute to a more pleasant life is directly related to the way families organize themselves to deal with adversity. Therefore, even though the diagnosis favors a decrease in the quality of life of caregivers, the way in which families create adaptation strategies and receive social support is of great relevance.

Considering the above that may be experienced by caregivers/parents of children with ASD, the importance of developing an attentive and welcoming approach to these individuals becomes evident. In this context, with an awareness of the importance of taking into account aspects related to the psychological well-being of caregivers of children with ASD, the hypothesis is raised that they present greater symptoms of anxiety and lower quality of life, and having higher levels of anxiety could worsen the perception of quality of life. Therefore, we sought to verify the relationship between the perception of anxiety symptoms and perceived quality of life among caregivers/parents of children on the autism spectrum in an assistance center in Belém, Amazon, Brazil.

## 2. Materials and Methods

### 2.1. Type of Study and Localization

This is a cross-sectional, descriptive, and analytical study, carried out from June to August 2022, at the Autistic Spectrum Disorder Assistance Center (NATEA). The description of the localization already appears in the study by Da Silva and Gomes [14], which is the first publication of a larger project on the eating behavior of children and adolescents with ASD and the anxiety and quality of life of their caregivers.

### 2.2. Participants

A convenience sample was compiled. With regard to inclusion and exclusion criteria, as presented in Da Silva and Gomes’s study [14], the following were selected: caregivers of children aged between 3 years and 11 years and 11 months diagnosed with ASD, who were monitored at CIIR; CIIR participants; and caregivers who agreed to participate in the research by signing the free and informed consent form.

Caregivers of children who did not have a confirmed diagnosis of ASD were excluded, as well as caregivers of children under 3 years old or over 12 years old; caregivers of twins with autism; those who did not attend CIIR; those who did not agree to participate in the study and did not sign the informed consent form; and parents or guardians who do not actively participate in their child’s care.

### 2.3. Ethical Aspects

This manuscript is part of a research project which, as described by Da Silva and Gomes [14], was approved by the Ethics and Research Committee of the Center for Tropical Medicine at the Federal University of Pará, under opinion number 5,354,653. This research was carried out in compliance with the legal requirements of Resolutions 466/12 and 510/16 of the National Health Council and the Declaration of Helsinki. We emphasize that all participants signed the free and informed consent form.

### 2.4. Instruments

A form containing questions on sociodemographic characteristics was applied through interviews, addressing questions on the type of caregivers (mother, father, grandmother), the age of the children (average), and the number of children with ASD (average).

To assess perceived quality of life, the Short Form Healthy Survey (SF-36) item was used, translated and validated into Portuguese by Ciconelli, Ferraz, Santos, Meinão, and Quaresma [15]. This inventory uses 36 questions on various aspects that may relate to health-related perception and quality of life. To analyze the results, the domains of this instrument were grouped into a physical component (CF), to assess how much physical limitations can interfere with an individual’s daily life, which grouped the domains of functional capacity, physical aspects, pain, and vitality (composed of four items that consider both energy and fatigue levels), and an emotional component (EC), to assess how much emotional changes can interfere with an individual’s life, which encompassed the domains of mental health, general health status, and emotional and social aspects.

Each category ranged from 2 to 10 items that can be summarized with the overall physical component score and the overall emotional component score. The results were expressed on a scale from 0 to 100 (obtained through Raw Scale calculation), where 0 corresponds to the worst perception and 100 to the best perception of quality of life.

To check anxiety symptoms, the Beck Anxiety Inventory (BAI) was applied, a symptomatic scale designed to measure the severity of anxiety symptoms. It consists of 21 items, in which the participant must score according to the symptoms that affect them, on a four (4)-point scale, which reflects the levels of increasing severity of each symptom: 1—“absolutely not”; 2—“slightly, it didn’t bother me much”; 3—“moderately, it was very unpleasant, but I could bear it”; and 4—“severely, I could hardly bear it”. The total score is the sum of the scores of the individual items, allowing classification into levels of intensity of anxiety symptoms, where the minimum level presents total scores from 0 to 7, the mild level presents total scores from 8 to 15, the moderate level presents total scores from 16 to 25, and the severe level presents total scores from 26 to 63 [16].

### 2.5. Statistical Analysis

The data were tabulated and analyzed using Statistical Package for the Social Sciences (SPSS), version 25.0. There were no missing data. The categorical variables were expressed as absolute frequency and proportion or were expressed as mean and standard deviation. The Chi-square test was applied to identify differences between categories of categorical variables; the Mann–Whitney Test to compare the scores of the quality of life domains according to the level of anxiety of the caregivers; the Spearman brightness test to verify bivariate consolidation; and those variables that were demonstrated in the Spearman test were entered into the multiple linear regression model to evaluate the predictors of the general emotional component of perceived quality of life. They were considered as a dependent variable (the general emotional component) and as co-variables (general anxiety score, child’s age (years), and number of children with ASD). For all analyses, a statistical significance level of *p* < 0.05 was considered.

## 3. Results

Eighty caregivers of children with ASD participated in this study. In 68.7%, mothers were the caregivers responsible for autistic children. The average age of the children was 6.9 ± 2.5 years and the average of the number of siblings with ASD was 1.2 (±0.4). Regarding perceived quality of life, the items with the lowest scores, on average, and which, therefore, demonstrated greater commitment on the part of caregivers, were, in order, (1) limitation due to physical aspects, (2) limitation due to emotional aspects, (3) vitality, and (4) pain. Regarding anxiety symptoms, of the total number of caregivers, 68.8% (*n* = 55) had a severe level of anxiety (Table 1).

Table 2 presents perceived quality of life data according to the level of anxiety in caregivers of children with ASD. We can verify that caregivers with severe anxiety levels had a worse perception of quality of life in the domains of pain (*p*-value = 0.012), social aspects (*p*-value < 0.001), limitation due to emotional aspects (*p*-value = 0.001), and mental health (*p*-value < 0.001), when compared to caregivers with a moderate level of anxiety. However, in terms of functional capacity, caregivers with a moderate level of anxiety had a better functional capacity score (*p*-value = 0.001).

Table 3 shows a positive correlation between the caregiver’s general anxiety score and the child’s age in years (ρ^2^ = 0.187; *p*-value = 0.049); a correlation was also observed between the number of children’s siblings with ASD and the general physical component of the caregiver’s perceived quality of life (ρ^2^ = 0.210; *p*-value = 0.031) and the general emotional component of the caregiver’s perceived quality of life (ρ^2^ = 0.211; *p*-value = 0.030). However, there was a negative correlation between the caregiver’s general anxiety score and the general physical component of the caregiver’s perceived quality of life (ρ^2^ = −0.213; *p*-value = 0.029) and the general emotional component of the perceived quality of life (ρ^2^ = −0.435; *p*-value < 0.001).

After bivariate correlation analysis, the variables that presented a significant result were included in the multiple linear regression model.

A correlation was observed between the general emotional component of perceived quality of life and the general anxiety score, which remained significant regardless of the child’s age and the number of children with ASD (B = −0.471; CI = −0.671; −0.274; *p* < 0.0001); that is, the level of anxiety is a predictor of the perceived quality of life of these caregivers, as shown in Table 4.

## 4. Discussion

The present study evaluated the relationship between the perception of anxiety symptoms and the perceived quality of life of caregivers/parents of children with ASD. It was observed that the majority of caregivers for children with autism were mothers, the average age of the children was 6.9 ± 2.5 years, and the average number of siblings with ASD was 1.2 (±0.4). This is similar to the study of Demsar and Bakracevic [17], in which the authors aimed to determine the level of stress, anxiety, and depression among Slovenian parents of children with autism spectrum disorder and their coping mechanisms, and they observed that most caregivers were mothers, the mean age of the children was 6.29 (±2.04), and most families had one (45.2%) or two (42.9%) children, while a smaller proportion (11.9%) had three.

The mother, as the child’s caregiver, is responsible for seeking treatment and for the daily care of her child, and as such, needs to make adaptations in her daily life that can result in the impoverishment of her social, emotional, and professional life, as well as consequent physical detriment [18]. In this context, the responsibility of the caregiver has been presented as an exhausting task, resulting in increased levels of anxiety and reduced quality of life for the caregiver.

According to Samadi and Samadi [19], the authors aimed to understand the potential of caregivers and the caregiving process for an individual with ASD, and mention that most studies carried out to identify the challenges associated with caring for a member with ASD in family environments focus mainly on quality of life and maternal stress, as mothers are generally the main caregivers of individuals with ASD in the family.

The results of the present study corroborate those of a study carried out by Asahar, Malek, and Isa [20], who demonstrated the prevalence of mother caregivers in their research. In the study by Traustadottir [21], the authors believed that in families with children with developmental disabilities, mothers are less likely to have paid employment and are therefore expected to have greater childcare responsibilities.

In the present study, severe anxiety levels were found in most caregivers, in addition to lower scores in the perceived quality of life domains, indicating deeper impairments in limitation due to physical aspects, vitality, and limitation due to emotional aspects measured by the scale. Furthermore, it was observed that caregivers with severe anxiety levels had a worse perception of quality of life in the domains of pain, social aspects, limitation due to emotional aspects, and mental health when compared to caregivers with a moderate level of anxiety. However, in terms of functional capacity, caregivers with a moderate level of anxiety had a better functional capacity score.

Ten Hoopen et al. [22] observed in their study on caregivers of children with autism that the lower quality of life related to the care of primary caregivers was associated, among other factors, with their own anxiety/depression, but also with more anxiety/depression in the affected children.

In a study by Öz, Yüksel, and Nasiroğlu [23], the authors aimed to evaluate the effect of ASD on the perception of internalized stigma, symptoms of depression and anxiety, and the quality of life of mothers of children with this disorder, and they observed that the mothers presented moderate symptoms of anxiety, and their quality of life scores were found to be low. The authors also observed that mothers whose children presented major difficulties specific to autism had higher levels of anxiety and a lower quality of life.

According to Patel et al. [24] and Akram et al. [25], the quality of life of these caregivers is greatly affected in the “physical health” and “psychological” domains, as they need to perform different functions in their lives to face the challenges of their children with ASD and, consequently, they become anxious, depressed, exhausted, and commonly frustrated while caring for their child. Patel et al. [24] also observed that parents who have children with ASD have physical health problems.

Turnage and Conner [26] carried out an integrated review of the literature on quality of life in parents of individuals with ASD, and observed that quality of life in the aspects of physical, psychological, and social health and spirituality was lower in parents of children with ASD compared to typical parents. The level of support for the child with ASD was considered a risk factor for the parents’ quality of life, and as a protective factor, the level of education of the parents and the level of support for the child’s ASD were observed.

Therefore, although the main reasons identified as causes of anxiety and reduced perceived quality of life have not been investigated in the present study, several factors involved in caring for a child with autism may be related. Tathgur and Kang [27] aimed to assess the concerns of caregivers of children with ASD and observed that caregivers face several challenges that negatively impact their physical health, psychological well-being, social reactions, and financial balance. Additionally, caregivers reported concerns related to the availability of ASD services and poorly coping with the diagnostics.

In the study by Papadopoulos [28], the author evaluated the experiences and challenges of parenting among mothers of children with autism in Greece, and observed that families with a child with ASD experience diverse challenges in many aspects, from emotional and family burden to social and financial burden.

According to Almeida et al. [29], the diagnosis of autism can cause possible impacts on the family environment, such as stressful situations and overloads. Therefore, it is suggested that, in the present study, factors such as having more than one autistic child, a child with feeding difficulties, exacerbated financial expenses, and deterioration of social and sometimes professional relationships may contribute to an increase in anxiety symptoms and reduced perceived quality of life.

A correlation was observed between the general emotional component of perceived quality of life and the general anxiety score, regardless of the child’s age and the number of children with ASD. In the study by Vernhet et al. [8], the authors observed that the perception of mothers and fathers on the impact on their quality of life was not associated with the age of their children.

Studies show that caregivers/parents of children with ASD generally present a greater loss in emotional health compared to typical families; this is because ASD presents characteristics that directly reflect on the family environment, such as low social interaction and an inability to relate to other people, resulting in behavioral problems and creating more prolonged stress due to daily care [30].

For Sprovier and Assumpção [31], the way a family deals with the condition is influenced by acceptance, interpretation, and the way the individual deals with the challenges to which they are subjected.

Carvalho-Filha et al. [32] observed results of this nature when analyzing the daily lives of caregivers of children with autism; they found that the majority of caregivers reported that their daily life is extremely focused on caring for the child with autism. The greatest difficulties were in relation to communication and feeding; in addition, the areas most affected in daily life were leisure and work.

The results show the need for an approach aimed not only at children, but also at their caregivers, as their perceived quality of life is compromised due to the daily care of children with ASD. According to Samadi and Samadi [19], it is important to involve parents in the implementation of treatment for children with ASD; however, different aspects of care must be understood and taken into consideration.

Furthermore, it is essential to raise community awareness about the particularities of the universe of individuals with ASD, in favor of more empathy, solidarity, spaces for listening, and support for the families of individuals with ASD.

Regarding the limitations of the present study, it can be mentioned that it is a cross-sectional study with non-probabilistic convenience sampling, and economic and demographic data of caregivers were not investigated. Furthermore, it is important to mention that individuals with ASD represent a very distinct group, as the spectrum includes different types of skills and challenges, which can directly impact caregivers. However, despite the limitations, the present study made it possible to know the psychological well-being of caregivers, contributing to reinforcing the need for patient- and family-centered care, to help caregivers develop positive coping strategies to deal with the problems they may encounter when caring for a child with autism, thus contributing to reducing anxiety symptoms and improving the perceived quality of life. Furthermore, no studies on this topic were found to have been carried out in the northern region of Brazil.

It is suggested that further research be carried out on this topic, with better investigation into the influence of the parental style and lifestyle of the child with ASD and their family members, such as screen time and physical exercise, in addition to considering the support level, the child’s social impairment, and parental knowledge about ASD, among others. Thus, it will be possible to contribute to a better quality of scientific evidence and cooperate in clarifying families who have children with ASD, in order to help in the development of care and therapeutic assistance programs for caregivers.

## 5. Conclusions

In the present study, the perception of high levels of anxiety and impairment in the perceived quality of life of caregivers of children with autism was observed, as well as a correlation between these aspects and characteristics of children with ASD. It was found that the higher the child’s age and the number of children with ASD, the higher the level of anxiety and the lower the perception of quality of these caregivers. In linear regression analysis, the anxiety symptom score is a predictor of caregivers’ perceived quality of life in emotional aspects.

Therefore, the need for multidisciplinary monitoring and patient- and family-centered care is highlighted. Early intervention approaches to improve children’s autism-specific difficulties can contribute to a positive effect on the children themselves, as well as alleviating anxiety symptoms in caregivers and consequently improving quality of life.

## Figures and Tables

**Table 1:** Type of caregivers, age of children, number of siblings, perceived quality of life, and level of anxiety symptoms of caregivers of children with autism spectrum disorder followed in a public assistance service in Amazon, 2022.

	*n* (%)/Average (±SD)
Type of caregivers	
Mother	55 (68.7)
Father	15 (18.8)
Grandmother	10 (12.5)
Age of children	6.9 (±2.5)
Number of siblings with ASD	1.2 (±0.4)
Perceived Quality of life	
Functional capacity	76.1 (±20.4)
Limitation due to physical aspects	38.4 (±30.0)
Pain	44.6 (±18.0)
General health status	52.9 (±10.1)
Vitality	41.8 (±15.0)
Emotional aspects	53.9 (±19.4)
Limitation due to emotional aspects	40.2 (±36.2)
Mental health	57.7 (±16.4)
Level of anxiety	
Minimum level	0 (0.0)
Light level	0 (0.0)
Moderate level	25 (31.3)
Severe level	55 (68.8)
Total anxiety score	39.7 (±15.5)

Chi-square test; ASD = autism spectrum disorder.

**Table 2:** Perceived quality of life according to the level of anxiety of caregivers of children with autism spectrum disorder followed in a public assistance service in Amazon, 2022.

Perceived Quality of Life	Moderate Level of Anxiety (*n* = 25)	Severe Level of Anxiety (*n* = 55)	*p*-Value
	Average (±DP)	Average (±SD)	
Functional capacity	85.6 (±17.5)	71.7 (±20.2)	0.001
Limitations due to physical aspects	48.0 (±35.3)	34.0 (±26.5)	0.168
Pain	37.5 (±17.9)	47.8 (±17.2)	0.012
General health status	54.0 (±10.5)	52.4 (±10.0)	0.258
Vitality	46.4 (±17.8)	39.7 (±13.2)	0.149
Social aspects	66.5 (±13.8)	48.2 (±19.0)	<0.001
Limitations due to emotional aspects	60.2 (±34.7)	31.1 (±33.3)	0.001
Mental health	72.3 (±11.3)	51.0 (±13.8)	<0.001

Mann–Whitney test.

**Table 3:** Correlation between level of anxiety symptoms and caregivers’ perceived quality of life and characteristics of children with autism spectrum disorder monitored in a public assistance service in Amazon, 2022.

Characteristics of Caregivers	Characteristics of Children with ASD	
	Age (Years)	Number of Siblings with ASD
General anxiety score		
ρ^2^	0.187	−0.033
*p*-value	0.049	0.385
General physical component of quality of life		
ρ^2^	0.129	0.210
*p*-value	0.128	0.031
General emotional component of quality of life		
ρ^2^	0.056	0.211
*p*-value	0.310	0.030
Characteristics of caregivers	Perceived quality of life	
	General physical component of quality of life	General emotional component of quality of life
General anxiety score		
ρ^2^	−0.213	−0.435
*p*-value	0.029	<0.001

Spearman correlation test; ASD = autism spectrum disorder.

**Table 4:** Multiple linear regression analysis between the emotional component of perceived quality of life and the general anxiety score of caregivers of children with autism spectrum disorder, monitored in a public assistance service in Northern Brazil, 2022.

	*B*	IC 95% (Minimum; Maximum)	*p*-Value
Model 1			
General anxiety score	−0.455	−0.658; −0.255	<0.001
Model 2			
General anxiety score	−0.485	−0.688; −0.285	<0.001
Child’s age	0.183	−0.110; 2.359	0.074
Model 3			
General anxiety score	−0.471	−0.671; −0.274	<0.0001
Child’s age	0.134	−0.435; 2.079	0.197
Number of children with ASD	0.190	−0.447; 14.824	0.065

Multiple linear regression. Dependent variable: general emotional component. Co-variables: general anxiety score, child’s age (years), and number of children with autism spectrum disorder. *B* = regression coefficient.

## Data Availability

The data are not publicly available as they contain the personal information of the participants involved. Therefore, the data of this work are confidential to maintain the privacy of those involved.
